# Changes in talocrural and subtalar joint kinematics of barefoot versus shod forefoot landing

**DOI:** 10.1186/s13047-014-0042-9

**Published:** 2014-10-14

**Authors:** Mako Fukano, Toru Fukubayashi

**Affiliations:** Faculty of Sport Sciences, Waseda University, 2-579-15, Mikajima, Tokorozawa, Saitama 359-1192 Japan

**Keywords:** Kinematics, Tibia, Talus, Calcaneus, Shoes

## Abstract

**Background:**

Synergetic talocrural and subtalar joint movements allow adaptation to different footwear and/or surface conditions. Therefore, knowledge of kinematic differences between barefoot and shod conditions is valuable for the study of adaptations to footwear conditions. The objective of this study was to assess the kinematic differences in the talocrural and subtalar joints during barefoot and shod landing.

**Methods:**

Seven healthy participants (4 males and 3 females) participated in a landing trial under barefoot and shod conditions. Fluoroscopic images and forceplate data were collected simultaneously to calculate the talocrural and subtalar joint kinematics and the vertical ground reaction force.

**Results:**

Upon toe contact, the plantarflexion angle of the talocrural joint during the barefoot condition was significantly larger than that during the shod condition (barefoot, 20.5 ± 7.1°, shod, 17.9 ± 8.3°, *p =*0.03). From toe contact to heel contact, the angular changes at the talocrural and subtalar joint were not significantly different between the barefoot and shod conditions; however, the changes in the subtalar eversion angles in the barefoot condition, from heel contact to 150 ms after toe contact, were significantly larger than those in the shod condition.

**Conclusions:**

These results suggest that footwear was able to reduce the eversion angle of the subtalar joint after heel contact during landing; the effect of wearing footwear was quite limited. Therefore, induced rearfoot kinematic alterations to prevent or manage injuries by neutral-type footwear are likely to be impractical.

## Background

The synergetic movements of the talocrural and subtalar joints, often referred to as the ankle joint, act to adapt foot contact with an uneven surface and to transmit the load between the foot and shank during weight bearing activities.

Sports footwear is a key component of the equipment used in sports and exercise, and acts as an interface between the foot and the ground to help protect the foot from injury. The kinematics of the lower extremity during walking and running can also be altered by footwear and orthotics. Previous studies have reported that footwear or foot orthotics are able to reduce rearfoot eversion [1] and/or tibial internal rotation [2] during running. Rearfoot motion, including the talocrural and subtalar joints, has been the subject of research into lower extremity pathology that has led to the concept that foot pronation control, by footwear, may prevent injury [3]. Some very recent research, however, has shown that motion control by footwear does not reduce the risk of injury [4-6]. Although controlling foot pronation has been practiced to manage various sports injuries, a consensus regarding the biomechanical effects of footwear has not been achieved.

Methodological disadvantages are common when measuring and interpreting talocrural and subtalar joint kinematics and the effects of footwear on these 2 joints. Modern motion analysis techniques that require arranging reflective markers on selected anatomical landmarks do not provide precise talocrural and subtalar kinematics because of skin movement artifact and the inability to access the talus [7,8]. Intracortical pins have been used to quantify the effects on tibiocalcaneal kinematics when wearing footwear [9], orthotics [10], and modified shoe soles [11] during slow running owing to their ability to provide precise *in vivo* bone movement determinations. However, this method is highly invasive and may influence movement because of the application of local anesthesia. Furthermore, in cases where the participant is wearing footwear, researchers must employ strategies, such as having the volunteers wear sandals with straps [12] or cutting openings in the footwear [13], to attach markers to selected points of skin that may otherwise be covered by the footwear. These strategies work, but may change the mechanical properties of the footwear, even if the openings are kept small to minimize the damage to the footwear.

In recent years, radiographic shape-matching techniques (i.e., 3 dimensional 2 dimensional (3D–2D) model-image registration) have been applied to the foot and ankle [14,15] to overcome the methodological difficulties associated with motion analysis due to skin movement artifact. Shape-matching techniques are advantageous, as they provide detailed information regarding *in vivo* joint kinematics with a less invasive methodology. Thus, this technique may be used to quantify 3D kinematics following total ankle arthroplasties [16,17] and in healthy ankles during dorsiflexion-plantarflexion activities [15] and simulated static gait positions [14]. This method also overcomes the problem of impaired footwear function by visualizing the actual foot bones. Although this technique has many advantages, the sampling frequencies (7.5–30 Hz) in previous studies were lower than those used in modern motion analysis techniques for evaluating quick movements. Therefore, to the authors’ knowledge, detailed quantification of talocrural and subtalar joint movement has not reported during one sequence of dynamic activities. Campbell et al. [18] demonstrated differences in rearfoot motion between barefoot and shod walking conditions using biplane fluoroscopic images to analyze the relative bone positions of the tibia and calcaneus; however, the talocrural and subtalar kinematics have not been clarified.

Thus, the present study quantitatively compared the kinematics of the talocrural and subtalar joints during barefoot and shod landings. The null hypothesis was that healthy participants would exhibit the same talocrural and subtalar joint kinematics during both barefoot and shod landings.

## Methods

This study was approved the Ethics Committees of the Graduate School of Sport Sciences (# 0085), Waseda University, Japan. Informed written consent regarding the purposes and procedures used in this study was obtained from each participant prior to their involvement in the study.

Seven healthy participants (4 males, 3 females) participated in this study (average age, 23.5 ± 1.6 years). All of the participants were free of lower extremity pain and did not have a history of serious injury or surgical treatment, and did not suffer from any subjective symptoms that interfered with sporting activities. When we conducted this experiment, each individual was participating in various recreational sports activities twice or thrice weekly, and none of them was a habitual barefoot and/or forefoot strike runner. We planned this study using repeated variables from matched pairs of study participants. A sample size calculation, based on prior normally distributed data, indicated that 6 participants were required to reject the null hypothesis (*p* <0.05, power =0.9).

**Figure 1:**
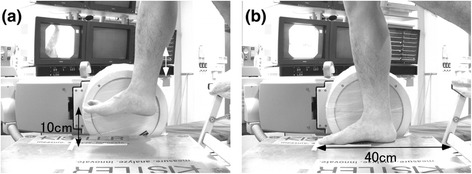
**Landing trial.** The participants’ preparatory positions **(a)** and landing positions **(b)** are shown.

The participants were required to conduct forefoot landings with their knee extended (Figure 1). Participants stood on a platform with their left leg and their right foot extended forward and placed 10 cm above from the surface of the force plate; the landing height was limited to 10 cm because of the recording height of the equipment. Further, a landing from an approximate height of 10 cm is a common daily activity, similar to that involved in marching-in-place during aerobic exercise or descending stairs. Participants shifted their centers of gravity forward and conducted a landing 40 cm ahead of the left toe on their right foot; participants were asked to stop and remain balanced after the landing. All participants were instructed on proper landing technique and were required to practice beforehand. The participants were required to conduct a landing, while barefoot and while wearing athletic footwear (Adidas Response Cushion; Adidas, Herzogenaurach, Germany). The footwear used in this study is marketed for beginner and mid-level runners and is considered neutral-type footwear; we did not modify the footwear. The midsole material was composed of ethylene vinyl acetate and the outsole material was carbon rubber. This footwear was chosen as the test footwear because of its wide use during exercises, including walking and running. Before the experiment, the lengths of each participant’s feet were measured, in the standing position, and footwear that was 1.5 ± 0.2 cm longer than the length of their right foot was selected as the experimental footwear. Participants were required to fasten their own shoelaces.

Each trial was recorded using lateral fluoroscopy. Images were obtained for 3 trials at a rate of 60 Hz, using 1-ms X–ray pulses (200 mA, 50 KV, 512 × 512 pixel images; Integris BH5000R.1, Philips, Amsterdam, Netherlands). A maximum sampling rate of 60 Hz was chosen because the fluoroscopy unit used in this study was a clinical examination device, specific to the cardiovascular system. Simultaneously, ground reaction force was measured at a rate of 1000 Hz using a force plate (Kistler 9286A; Kistler, Winterthur, Switzerland). The time histories of two different sample frequency data were synchronized using custom-written programs (MATLAB, MathWorks, Natick, MA, USA).

**Figure 2:**
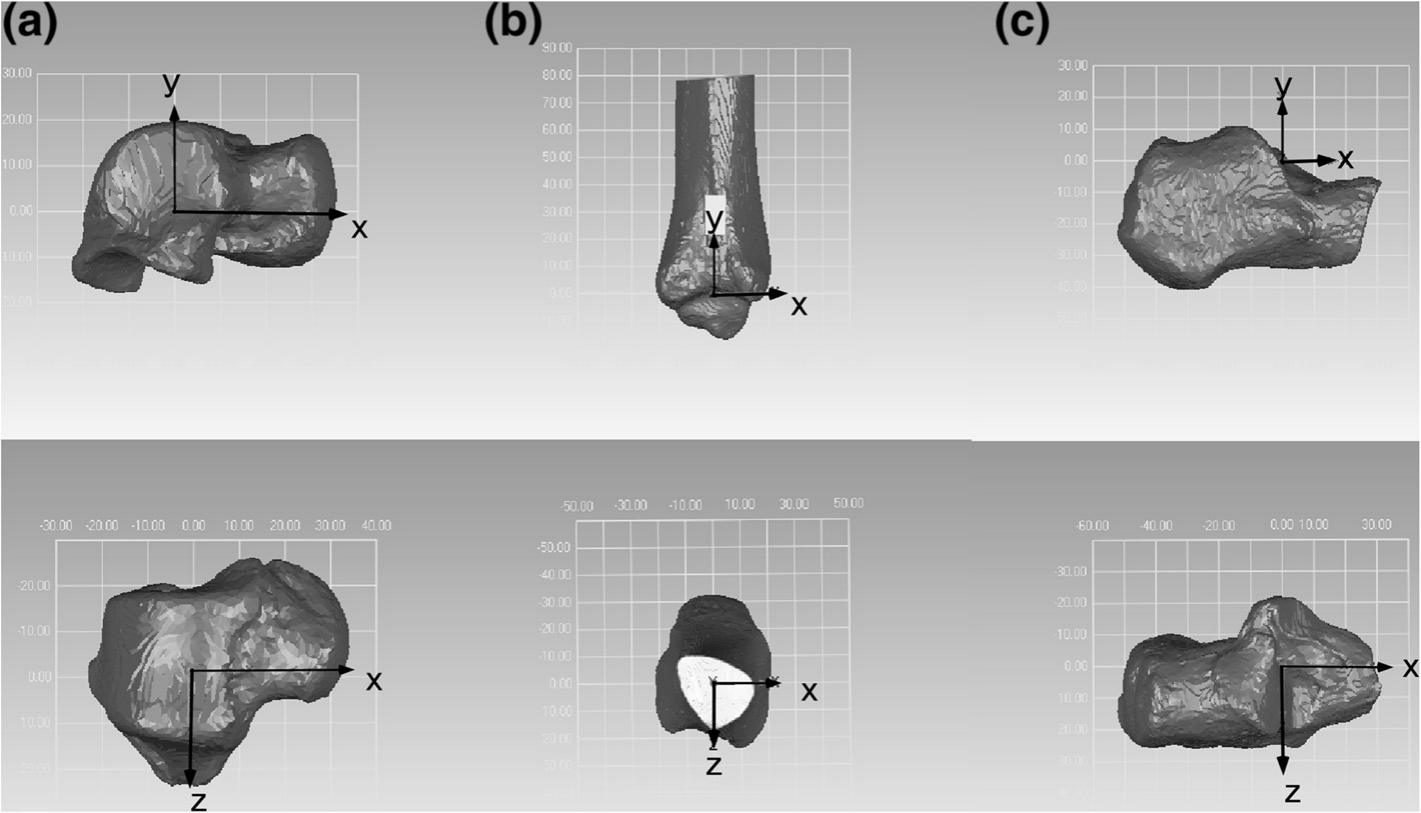
**Anatomic coordinate system of the talus (a), tibia (b), and calcaneus (c).**

Geometric bone models of the tibia, talus, and calcaneus were created from computed tomography (CT) scans (IDT 16, Philips, Amsterdam, Netherlands) of the right leg. The CT scans also used image areas of 512 × 512 pixels and a 0.4-mm overlapping slice thickness, spanning the distance 15 cm proximal to the malleolus to the plantar surface (200 mA/slice, 120 kV, CTDI 15.5 mGy). Exterior cortical bone edges were segmented using open source software (ITK-SNAP [19]). Anatomical coordinate systems were embedded in each bone model, according to published definitions [15,20] (Geomagic Studio, Geomagic, Morrisville, NC, USA). The tibial origin was placed at the centroid of the tibial plafond; the Y-axis was parallel to the shaft; and the X-axis was the line perpendicular to the line connecting the anteromedial and anterolateral edges of the tibial plafond. The talar origin was placed at the center of a circle that circumscribed the trochlea tali; the circle contained the midpoint of the anteromedial and anterolateral edges and the midpoint of posteromedial and posterolateral edges of the trochlea. The Z-axis was defined as the line perpendicular to the circle, with the X-axis parallel to the line that linked the anterior and posterior edges of the trochlea with the circle. The calcaneal origin was placed at the center of the line that linked the most lateral point of the posterior articular surface with the most medial point of the middle articular surface. The X-axis was parallel to the inferior surface of the calcaneus, and the Y-axis was parallel to the lateral surface of the calcaneus (Figure 2). The motion at the talocrural joint was defined as the motion of the talus relative to the tibia. The motion at the subtalar joint was defined as the motion of the calcaneus relative to the talus. Kinematics at the talocrural and subtalar joints were expressed using a joint coordinate system because it provides an anatomic, easily interpreted description. We also defined terms to describe the motion of the talocrural and subtalar joints: dorsi/plantarflexion was defined as the rotation around the mediolateral axis, eversion/inversion was defined as rotation around the anteroposterior axis, and external/internal rotation was defined as the rotation around the proximodistal axis.

**Figure 3:**
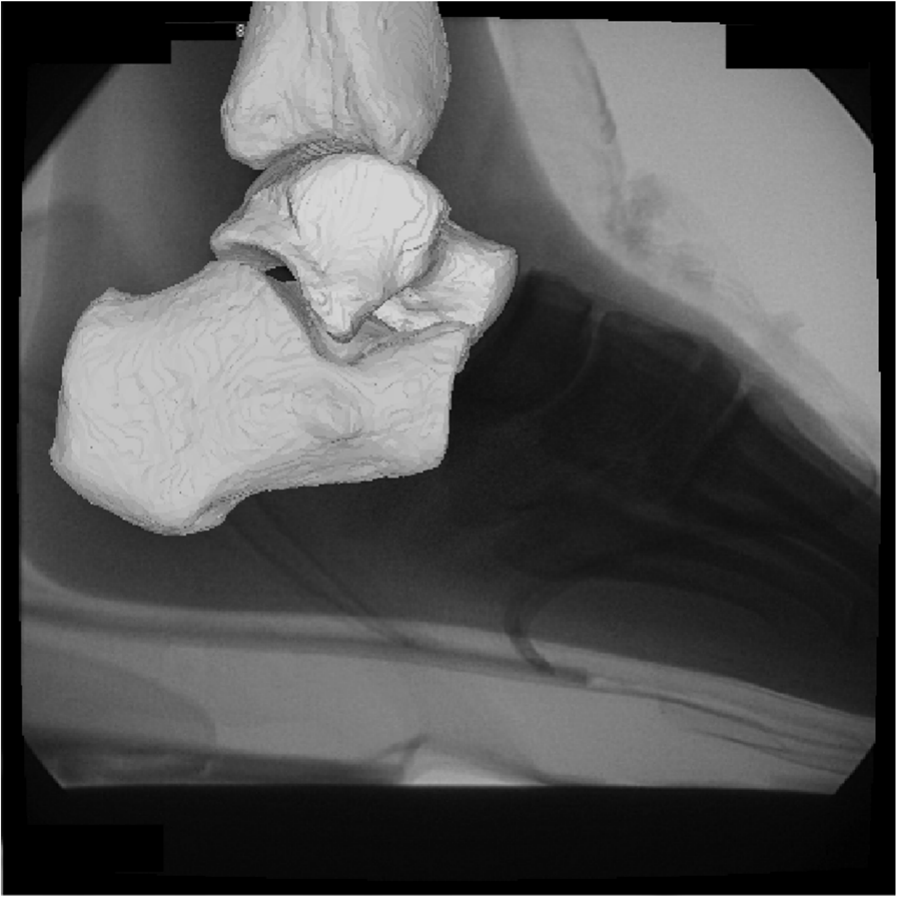
**Three-dimensional two-dimensional model-based registration technique.** (JointTrack, http://sourceforge.net/projects/jointtrack/).

Three-dimensional bone positions and orientations upon landing were determined using a 3D-2D model-based registration technique that uses bone models and single-plane fluoroscopic images [21] (JointTrack, http://sourceforge.net/projects/jointtrack/). The created bone models were projected on the distortion-corrected fluoroscopic images and were precisely adjusted, frame-by-frame, until the counter of the projected bone models matched the osseous counter in the fluoroscopic images (Figure 3). We considered the best matching contours were generated when both the overlapping all inflexion points and the conforming curves of bone models and osseous contours matched. The data were analyzed using custom-written programs (MATLAB, MathWorks, Natick, MA, USA) using standard conventions [22]. One investigator repeated this matching protocol 3 times to maintain a high operating accuracy. Measurement repeatability was assessed by repeatedly (3 times) analyzing three images, with the average differences from the mean of 0.60 mm for in-plane translations, 1.8 mm for out-of-plane rotations, and 0.59° for rotations. The intraclass correlation coefficients (ICCs) for the kinematics data were >0.98 for dorsi/plantarflexion, >0.78 for eversion/inversion, and >0.91 for external/internal rotation.

The static reference position of the right foot of each participant was obtained before the trial with each participant standing the right leg. We analyzed kinematic data from 33 ms before toe contact to 250 ms after toe contact.

A paired *t*-test was used to compare the values between the barefoot and shod conditions; a 2-tailed test was used to test the null hypotheses. Significance was set at *p* <0.05.

## Results

**Figure 4:**
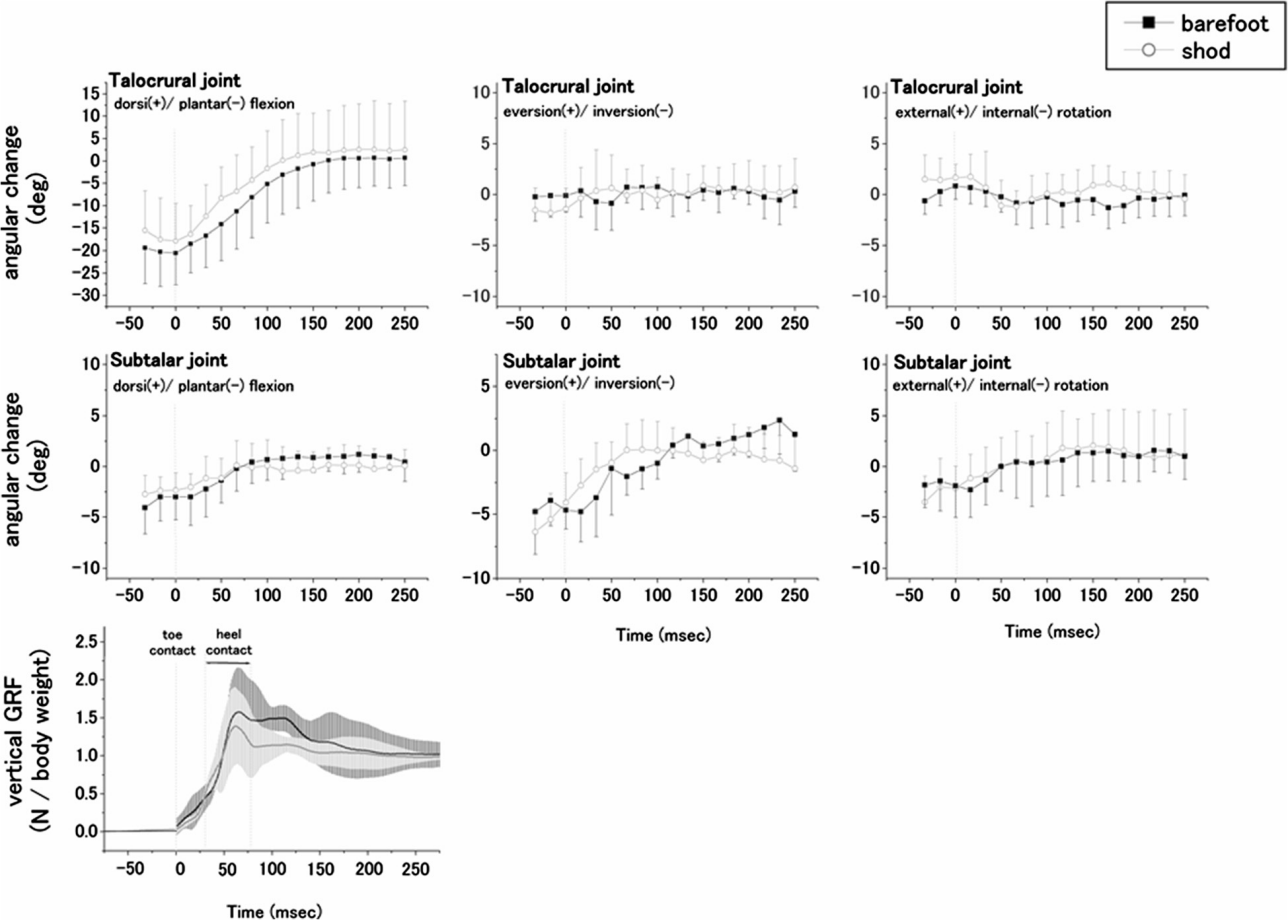
**Kinematic averages and standard deviations of the talocrural and subtalar joints, and the vertical ground reaction force (GRF) during landing.**

Figure 4 demonstrates the time course changes in the talocrural and subtalar joint angles and the vertical ground reaction forces determined during landing in both the barefoot and shod conditions. All participants demonstrated a forefoot landing. For the talocrural joint, the main motion after touchdown was dorsiflexion in both the barefoot and shod conditions. For the subtalar joint, the joint tended to be dorsiflexed, with eversion and external rotation after toe contact.

Upon toe contact, the talocrural joint position was slightly plantarflexed during both the barefoot and shod tests and the angle of the plantarflexion in the barefoot condition was significantly larger than that in the shod condition (*p* =0.008). With regard to the observed eversion/inversion and external/internal rotation, the talocrural joint position was not significantly different between the 2 conditions (Table 1).

**Table 1:** **Angles of the talocrural and subtalar joints at the time of toe contact**

**Joint**	**Condition**	**Barefoot**		**Shod**		**Mean difference**	**Difference**	***p value***
	**Variable**	**Mean**	**(SD)**	**Mean**	**(SD)**			
Talocrural joint	Dorsi(+)/plantar(−) flexion (deg)	−20.5	(7.1)	−17.9	(8.3)	−2.6	*	*0.008*
	Eversion(+)/inversion(−) (deg)	0.1	(1.6)	−1.4	(2.0)	−1.5	-	*0.071*
	External(+)/internal(−) rotation (deg)	0.8	(1.3)	1.7	(1.3)	0.9	-	*0.393*
Subtalar joint	Dorsi(+)/plantar(−) flexion (deg)	−3.0	(2.3)	−2.3	(1.7)	−0.7	-	*0.231*
	Eversion(+)/inversion(−) (deg)	−4.7	(1.5)	−4.1	(2.3)	−0.6	-	*0.111*
	External(+)/internal(−) rotation (deg)	−2.2	(2.2)	−1.9	(3.1)	−0.3	-	*0.471*

*, significant difference; SD, standard deviation.

From the time of toe contact until heel contact, significant differences in the angular changes for the 3 axial rotations were not observed between the 2 conditions. From the time of heel contact to 150 ms after toe contact, the angular changes in the eversion of the subtalar joint, in the barefoot condition, were significantly larger than those in the shod condition (*p* =0.044). The changes in the dorsi/plantarflexion and external/internal rotations were not significantly different between the 2 conditions (Table 2).

**Table 2:** **Talocrural and subtalar joint angle changes during each period after toe contact**

**Period**	**Joint**	**Condition**	**Barefoot**		**Shod**		**Mean difference**	**Difference**	***p value***
		**Variable**	**Mean**	**(SD)**	**Mean**	**(SD)**			
Toe contact - heel contact	Talocrural joint	Dorsi(+)/plantar(−) flexion (deg)	9.8	(9.2)	9.0	(5.0)	0.8	-	*0.763*
		Eversion(+)/inversion(−) (deg)	−0.9	(1.0)	2.1	(1.7)	−3.0	-	*0.076*
		External(+)/internal(−) rotation (deg)	−1.5	(1.2)	−2.6	(2.1)	1.1	-	*0.083*
	Subtalar joint	Dorsi(+)/plantar(−) flexion (deg)	2.0	(1.4)	1.7	(1.3)	0.3	-	*0.494*
		Eversion(+)/inversion(−) (deg)	1.8	(1.0)	3.0	(0.9)	−1.2	-	*0.083*
		External(+)/internal(−) rotation (deg)	2.1	(0.9)	3.5	(1.8)	−1.4	-	*0.197*
Heel contact - 150 msec after toe contact	Talocrural joint	Dorsi(+)/plantar(−) flexion (deg)	10.0	(1.9)	10.8	(3.1)	−0.8	-	*0.431*
		Eversion(+)/inversion(−) (deg)	1.4	(0.7)	1.7	(1.4)	−0.3	-	*0.412*
		External(+)/internal(−) rotation (deg)	−2.2	(1.9)	2.4	(1.4)	−4.6	-	*0.664*
	Subtalar joint	Dorsi(+)/plantar(−) flexion (deg)	2.2	(2.1)	0.7	(0.4)	1.5	-	*0.074*
		Eversion(+)/inversion(−) (deg)	3.2	(2.3)	0.7	(0.7)	2.5	*	*0.044*
		External(+)/internal(−) rotation (deg)	2.5	(2.2)	2.1	(2.2)	0.4	-	*0.769*
Maximum vertical ground reaction force (N/body weight)			1.58	(0.58)	1.39	(0.54)	0.2	*	*0.031*
							0.0		

*, significant difference; SD, standard deviation.

## Discussion

Athletic footwear design is based on various concepts in consideration of the protection of the human foot; however, knowledge of the effects of footwear on internal foot and ankle biomechanics is limited. The 3D-2D model-based registration technique, combining fluoroscopic images with CT, permits direct measurement of *in vivo* talocrural and subtalar joint kinematics while wearing footwear, without altering the footwear’s construction. The results of the present study suggest that wearing the footwear used in this study had limited effects on the talocrural and subtalar joint kinematics during landing. The null hypothesis that healthy participants would exhibit the same talocrural and subtalar joint kinematics during barefoot and shod landing was partially rejected.

At the time of toe contact, the talocrural joint showed greater plantarflexion in the barefoot condition than when the footwear was worn. This larger plantarflexion may be considered as a preparatory position against direct touchdown impact on the plantar surface. De Wit et al. [23] observed kinematic and plantar pressure differences during barefoot and shod running. In their study, more horizontal foot placements were observed during the barefoot condition, with more ankle plantar flexion, than during the shod condition; the flatter foot placement and lower peak heel pressures were also correlated. Hence, they concluded that the lower extremity joint configuration at touchdown, was prepared during free-flight, implicating an actively induced adaptation strategy during barefoot running. The authors also assumed that this adaptation limited the local pressure underneath the heel. Therefore, the greater plantarflexion at toe contact, while barefoot, might be the result of joint positioning in anticipation of foot-ground impact and corresponding the heel pain.

The angular change of eversion at the subtalar joint, between heel contact and 150 ms after toe contact, was decreased while wearing footwear. The reduced eversion resulted mainly from the midsole construction. Cheung et al. [24] reported a midsole structure comprising two materials; in their meta-analysis, a softer lateral and a firmer medial component construction was more effective for controlling calcaneal eversion than was heel flare or wedge modification. The reduced eversion was also attributed to the heel counter of the footwear; its primary function is to restrict overpronation (often used as an expression to indicate excessive complex movement during calcaneal dorsiflexion, eversion, and external rotation). Van Gheluwe et al. [25] stated that heel fit affects the eversional behavior of the calcaneus during ground contact, and that running shoes equipped with a rigid heel counter improved rearfoot control. People with excessive foot pronation, which, in the absence of clinical consensus, is generally considered to be a greater than normal excursion of calcaneal eversion, have a higher incidence of injuries, such as plantar fasciitis [26] and medial tibia stress syndrome [27]. Wang et al. [28] showed that foot eversion increased the intra-articular pressure, shifted the contact area to the anteroinferior aspect of the posterior articulation, and reduced the contact area upon anterior articulation contact in the subtalar joint. Lersch et al. [29] demonstrated that changes in the frontal plane position of the calcaneus resulted in higher strain differences within the Achilles tendon than did variations of the triceps surae muscle force ratios. Although their findings from cadaveric studies do not directly prove clinical relevance, wearing footwear may cause some changes in the force in some tissues at intrinsic and extrinsic ankle by altering subtalar eversion. Regarding the kinetics of the proximal joint, Eslami et al. [12] reported that the magnitude of rearfoot eversion was positively correlated with the peak knee adduction moment during shod running (or when wearing orthoses) in healthy volunteers. When considering injury pathology and/or prevention, the effects of motion control footwear should be examined, since many types of motion control footwear are marketed for people with overpronation.

The primary motion at the talocrural joint was dorsiflexion while the subtalar joint motion was complex, combining calcaneus dorsiflexion as well as eversion and external rotation during landing in both the barefoot and shod conditions. The movement pattern of the talocrural joint was consistent with previous *in vivo* fluoroscopic studies [14,17]. On the contrary, subtalar joint eversion, after heel contact, was reduced in the shod condition. A recent study reported that the subtalar joint moves in three planes with similar magnitudes since the rotation axis of the calcaneus runs obliquely in 3 dimensions [20]. Goto et al. [30] observed subtalar joint kinematics during dorsi-plantarflexion and demonstrated that the subtalar joint is essentially a uniaxial joint and the axis of the calcaneus relative to the talus runs antero-dorso-medially to postero-planto-laterally, penetrating the talar neck. The reduction of eversion also potentially alters the relative positions of the talus and, thus, the calcaneal rotation axis. Additionally, both the talocrural and subtalar joints had respective kinematic changes at different times during the shod landings. Our results suggest that each talocrural and subtalar joint has a different way of adaptating to footwear conditions.

There are some limitations to this study, including the interpretation of landing studies from a 10 cm height due to the spatial limitation. The maximum vertical ground reaction forces for the participants were approximately 1.4 times their body weight, which may be comparable to that occurring while stepping or descending low stairs, but less than the impact force occurring during competitive sports activities. An additional limitation involves the relatively small sample size. Since this study involved radiation exposure, the number of participants was kept to a minimum based on the sample size estimation. Moreover, different types and designs of footwear such as motion control shoes must be studied in this regard.

## Conclusions

Footwear was able to reduce the eversion angle of the subtalar joint after heel contact during forefoot landing, but the change was small; for this reason, the effect of wearing footwear was quite limited. Therefore, although controlling foot pronation has been practiced prevent or manage injuries, induced rearfoot kinematic alterations by neutral-type footwear may to be impractical. Future research should verify the efficacies of wearing different types and designs of footwear for rearfoot motion control.
